# Linkages between the molecular composition of dissolved organic matter and soil microbial community in a boreal forest during freeze–thaw cycles

**DOI:** 10.3389/fmicb.2022.1012512

**Published:** 2023-01-09

**Authors:** Yan Yang, Shulan Cheng, Huajun Fang, Yifan Guo, Yuna Li, Yi Zhou, Fangying Shi, Karen Vancampenhout

**Affiliations:** ^1^Key Laboratory of Ecosystem Network Observation and Modeling, Institute of Geographic Sciences and Natural Resources Research, Chinese Academy of Sciences, Beijing, China; ^2^College of Resources and Environment, University of Chinese Academy of Sciences, Beijing, China; ^3^Northwest Plateau Institute of Biology, Chinese Academy of Sciences, Xining, China; ^4^The Zhongke-Ji’an Institute for Eco-Environmental Sciences, Ji’an, China; ^5^Division of Forest, Nature and Landscape, Department of Earth and Environmental Sciences, Faculty of Sciences, KU Leuven, Leuven, Belgium

**Keywords:** freeze–thaw cycles, dissolved organic matter, pyrolysis gas chromatography–mass spectrometry, high-throughput sequencing, boreal forest

## Abstract

Soil dissolved organic matter (DOM) plays a vital role in biogeochemical processes. Global warming leads to increased freeze–thaw cycles (FTCs) in boreal forest soils, which can change DOM production and consumption. However, the interactions between the chemical composition of DOM molecules and the microbial communities that drive C decomposition in the context of freeze–thaw are poorly understood. Here, a FTCs incubation experiment was conducted. Combined with pyrolysis gas chromatography–mass spectrometry and high-throughput sequencing techniques, the relationships between DOM chemodiversity and microbial community structure were assessed. Results indicated that both low-frequency (2FTCs) and high-frequency freeze–thaw cycles (6FTCs) significantly increased soil dissolved organic carbon (DOC) contents in the surface (0–10 cm) and subsurface (50–60 cm) soil layers. In the topsoil, FTCs significantly reduced the relative abundance of aromatic compounds, but increased the relative proportions of alkanes, phenols, fatty acid methyl esters (Me) and polysaccharides in the DOM. In the subsuface soil layer, only the relative abundance of Me in the 6FTCs treatment increased significantly. The response of bacterial communities to FTCs was more sensitive than that of fungi, among which only the relative abundance of *Gammaproteobacteria* increased by FTCs. Moreover, the relative abundance of these taxa was positively correlated with the increment of DOC. Co-occurrence networks confirmed DOM-bacterial interactions, implying that specific microorganisms degrade specific substrates. At class level, *Gammaproteobacteria* were significantly positively correlated with labile C (polysaccharides and alkanes), whereas other bacterial classes such as *Actinobacteria*, *Alphaproteobacteria*, and *Thermoleophilia* were significantly positively correlated with aromatic compounds in the topsoil. Collectively, FTCs tended to activate DOM and enhance its biodegradability of DOM, potentially hampering DOC accumulation and C sequestration. These findings highlight the potential of DOM molecular mechanisms to regulate the functional states of soil bacterial communities under increased FTCs.

## Highlights

- Freeze–thaw cycles (FTCs) increase DOC release and change the chemical structure of DOM in the surface and deep soils in the boreal forest.
- The improvement of DOM bioavailability is detrimental to DOC accumulation.
- *Gammaproteobacteria* play a dominant role in DOC production under the scenarios of intensified freezing–thawing.

## 1. Introduction

Dissolved organic matter (DOM) is the most active and bioavailable component of organic matter in soil. It is defined as a heterogeneous continuum of organic molecules of various sizes, that are soluble in water and can pass through a 0.45 um pore size filter (Rombolà et al., 2022). Soil DOM is mainly composed of amino acids, polysaccharides, organic acids, and low molecular weight components (Caricasole et al., 2010). Although accounting for only 2% of total soil organic matter (SOM), DOM plays a central role as a microbial substrate source, in soil aggregation, in carbon storage, and in the supply of plant nutrients (Nebbioso and Piccolo, 2013). The composition and quantity of DOM in soils is susceptible to many natural and anthropogenic factors, microbial processes and soil properties (e.g., soil pH, C/N ratio). It is likely that the complex chemical composition of soil DOM is stongly affected by environmental variations and soil composition. The release of DOM due to these environmental factors may chemically alter soil nutrient cycles, as well as transport carbon with pore water through leaching and surface run-off, leading to carbon losses. Soil freeze–thaw cycles (FTCs) are a prominent aspect of global change in high-latitude ecosystems, and have a significant effect on soil DOM release and chemical changes (Gao et al., 2021).

Reduced snow cover at high latitudes leads to an increase in the frequency and severity of soil FTCs (Gao et al., 2021). FTCs cause repeated fluctuations in the soil water phase and in soil temperature. These changes lead to microbial cell death, soil aggregate disruption and exposure of exchange sites, which in turn leads to increases in soil DOM concentration, thus affecting sequestration and stability of SOC (Oztas and Fayetorbay, 2003; Tan et al., 2014). In the past decade, most of the research on DOM in the context of freeze–thaw was limited to the amount of DOM (Hentschel et al., 2008; Watanabe et al., 2019). Only a few studies have reported that freeze –thaw changed the chemical composition of DOM. They found that FTC reduced the polysaccharide content and increased the lignin content of DOM in forest soils (Schmitt et al., 2008; Wu et al., 2017).

Laboratory incubation studies furthermore indicated that 4–93% of soil derived DOM can be decomposed by microorganisms (Kalbitz et al., 2003). So, a more comprehensive understanding of DOM chemical composition is necessary to identify the organic compounds that control the susceptibility of DOM to microbial degradation (Ward and Cory, 2015). Among analytical techniques for determining DOM chemistry, pyrolysis–gas chromatography/mass spectrometry (Py-GC/MS) is an effective tool that can directly offer information on molecular structures (Rombolà et al., 2022). This technique adopts a thermal pulse method to break macromolecules into fragments which are suitable for GC. The technique is semiquantitative. Due to selectivity of the GC column and the large number of compounds after pyrolysis, it is difficult to use specific internal standards for quantitative analysis. Nevertheless, it can be used for assessing changes in the relative abundance of different macromolecular components in DOM (Kaal et al., 2017).

Soil microorganisms, in particular, are vital mediators of degrading organic matter and together with DOM biodegradability partly determine biogeochemical fluxes (Ward and Cory, 2015). Furthermore, specific compound metabolism has been linked to specific microbial groups (e.g., lignin decomposition, nitrogen fixation; Cottrell and Kirchman, 2000). That linkage implies that the microbiota in the natural environment have the ability to selectively use different carbon substrates (Judd et al., 2006). For instance, *copiotrophic* bacteria (e.g., *Proteobacteria*, *Acidobacteria*) tend to favor decomposition of protein components in DOM (Yang et al., 2020), whereas soil bacterial activity can be inhibited by high concentrations of organic acids (Lehmann et al., 2020). Fungi also play an important role in DOM degradation due to their broad enzymatic capabilities and substrate preferences (Glassman et al., 2018). Fungi are regarded as the main organisms producing DOM, because their activity results in an incomplete degradation of SOM (Zsolnay, 2003). FTCs improve C and N availability, i.e., they cause significant increase in dissolved organic carbon (DOC) and dissolved organic nitrogen (DON) contents, thereby affecting the composition and function of the soil microbial community (Feng et al., 2007). Preferential utilization of labile C and N by surviving microorganisms can alter decomposition and substrate preferences, enabling a shift from complex plant polymers to low molecular weight compounds found in necromass (Perez-Mon et al., 2020). The fate of DOM-microbe interactions in freeze–thaw environments has not been elucidated. Except for the physical release of DOM, it is necessary to know which particular microbial assemblages dominate DOM production and which microbiota are reduced.

The objectives of this study are to explore the changes in DOM chemical composition under FTCs treatments, as well as the microbial degradation mechanism and their controlling factors. We hypothesize that: (1) FTCs can increase the bioavailability of DOM and can promote the conversion of aromatic compounds into polysaccharides; (2) FTCs increase the activity of dominant species and thus increase mineralization, while eliminating sensitive microorganisms, both of which jointly dominate DOC production.

## 2. Materials and methods

### 2.1. Study area

The Greater Khingan Mountains in Inner Mongolia (Northeast China), are located at the southern edge of the Eurasian permafrost region. This region is characterized by a cold temperate continental monsoon climate with long cold winters and short warm summers, and a fragile permafrost that is sensitivity to global warming. The annual average air temperature is −5.4°C and the mean annual precipitation is 580 mm. The annual average temperature has been increasing 0.32°C/ 10a over the past 60 year (1960–2020; Liu et al., 2020). Compared with the daily average temperature during the spring freeze–thaw period in the past 20 years (2000–2020), the frequency of FTCs increased significantly (Supplementary Figure S1a). The study sites were selected in the Greater Khingan Mountains Forest Ecosystem Research Station (121^°^30′-121^°^31′ E, 50^°^49′-50^°^51′ N, altitude 800–1,000 m). The zonal vegetation is mainly composed of Dahurian larch (*Larix gmelinii*) mixed with a number of White birches (*Betula platyphylla*) in the arbor layer, with dense *Rhododendron dauricum* and *Ledum palustre* in the herbaceous layer (Gao et al., 2019). The dominant soil types are Cambic and Leptic Umbrisols (WRB; Lützow et al., 2006). The average thickness of the organic layer is 10 cm and the average thickness of the active layer is about 60 cm. The soil is slightly acidic, with a pH in the range 6.10–6.52 (Table 1).

**Table 1:** Soil properties under freeze–thaw treatment at different depths.

Soil layer	Treatment^†^	TC	TN	C/N	NH_4_^+^-N	NO_3_^−^-N	pH
		(g kg^−1^)	(g kg^−1^)		(mg kg^−1^)	(mg kg^−1^)	
0–10 cm	CK	130.67 ± 1.69	7.04 ± 0.06	18.57 ± 0.11	10.22 ± 0.70	0.42 ± 0.05	6.52 ± 0.25
	2FTC	128.49 ± 0.22	6.93 ± 0.22	18.58 ± 0.59	9.54 ± 1.53	0.22 ± 0.05	6.25 ± 0.10
	6FTC	127.37 ± 1.07	7.37 ± 0.17	18.18 ± 0.84	11.95 ± 0.56	0.44 ± 0.14	6.11 ± 0.05
	*F*	2.09	1.89	0.15	1.47	1.77	1.74
	*P*	0.2	0.23	0.86	0.3	0.25	0.25
50–60 cm	CK	164.88 ± 2.79	8.00 ± 0.08	20.61 ± 0.17	10.25 ± 0.47	0.36 ± 0.04a	6.10 ± 0.02
	2FTC	161.42 ± 3.00	7.42 ± 0.03	21.74 ± 0.45	6.51 ± 0.75	0.05 ± 0.02b	6.18 ± 0.03
	6FTC	166.21 ± 0.99	7.78 ± 0.21	21.39 ± 0.53	9.64 ± 2.55	0.08 ± 0.03b	6.15 ± 0.05
	*F*	1.03	4.86	1.98	1.66	10.64	1.21
	*P*	0.41	0.06	0.22	0.27	0.01	0.36

† CK, 2FTC, 6FTC are cultured at 5°C, freeze–thaw treatment of 2 cycles and 6 cycles, respectively. TC, total carbon; TN, total nitrogen. Lowercase letters represent significant difference among freeze–thaw treatments at each soil depth in one-way ANOVA (*p* < 0.05).

### 2.2. Soil sampling and FTCs experimental design

In September 2020, three 20 m × 20 m square plots were established in mixed forests dominated by White birch and Dahurian larch. Plot were separated by a buffer zone of 20 m wide. In each plot, after removing the litter layer, the soils were sampled at 10 points along the diagonal line at the depths of 0–10 cm and 50–60 cm using a soil auger (Φ = 4 cm). The sample soils from each plot at the same depth were mixed uniformly into a composite sample. Those samples were sieved with a 2-mm mesh to remove roots, gravels, etc. and subsamples were air-dried at 40°C to determine pH, soil moisture, total nitrogen (TN), and total carbon (TC). The remaining subsamples were used to conduct a simulation FTCs experiment.

The daily air temperature measured at the Forest Ecosystem Research Station in the Greater Khingan Mountains showed that freeze–thaw mostly occurs from mid-March to mid-April. The average temperature during that period range from −10°C to 5°C, and the number of freeze–thaw cycles in that period varies between 2 and 6 (Supplementary Figure S1b). Accordingly, the simulation FTCs experiment was designed with three treatments: (i) constant culture at 5°C (CK), (ii) two freeze–thaw cycles (2FTCs), and (iii) six freeze–thaw cycles (6FTCs). The incubation temperature was set to −10°C, −5°C, 2°C, and 5°C in sequence for one freeze–thaw cycle (Supplementary Figure S1c). Each temperature was cultured for 6 days and 2 days under the scenarios of 2FTCs and 6FTCs, respectively. Homogenized soil samples of 100 g each were put into 250 mL glass Mason jars with the moisture adjusted to 60% of water filled pore space (WFPS). All samples were pre-incubated at 5°C for 7 days to allow microorganisms to acclimatize. All samples were incubated for 55 days.

### 2.3. Measurement of soil chemical properties

A subsample of each air-dried soil sample was milled with a ball mill (MM400, Retsch GmbH, Haan, Germany) and then analyzed for soil TC and TN concentrations using an elemental analyzer (Vario EL III, Elementar, Hanau, Germany). Fresh soil samples were extracted with 2.0 M KCl (soil: solution = 1:10 w/v) and inorganic nitrogen (NH_4_^+^-N, NO_3_^−^-N) concentrations were measured using a continuous flow autoanalyzer (AA3, SEAL, Germany). Water-extractable DOC was determined using a TOC analyzer (Liqui TOCII, Elementar, Germany). Soil pH (soil: water = 1:2.5 w/v) was determined using a portable pH meter (Mettler Toledo FE28, Switzerland).

### 2.4. Molecular characterization of DOM by analytical pyrolysis

Pyrolysis-gas chromatography–mass spectrometry analysis was conducted for DOM characterization using a multi-shot pyrolyzer (PY-3030D, Frontier Laboratories, Fukushima, Japan) attached to an Agilent 7,890 N gas chromatograph (GC) connected to an Agilent 7000B mass spectrometer (MS). The GC was equipped with an elastic Quartz Capillary Column (HP-5MS, 30 m × 0.25 mm × 0.25 μm inner diameter). DOM-containing extracts were lyophilized using a freeze-dryer (ALPHA1-4/Ldplus, Germany). Lyophilized samples (10 mg) were weighted into a small stainless-steel cup and inserted into a pre-heated furnace. The pyrolysis temperature was set as follows: 50°C for 1 min then rose to 600°C, from 50°C to 250°C at a rate of 50°C min^−1^ and 250°C to 600°C at 30°C min^−1^. The GC oven was heated from 40°C to 290°C at 4°C min^−1^. The MS was operated in electron ionization mode (70 eV, scanning 50–550 m/z) with GC injector at 230°C and ion source temperature at 280°C. The carrier gas was helium (1.2 mL min^−1^). The relative proportion of each compound was equal to the percentage of the peak area of each product to the total peak area.

### 2.5. DNA extraction, amplification, and Miseq sequencing

Genomic DNA was extracted from 0.25 g of homogenized soil sample, using a PowerSoil DNA Isolation Kit (MoBio Laboratories, Inc., Carlsbad, CA, USA) according to the manufacturer’s recommendation. The V3-V4 regions of bacterial 16S rRNA were amplified using the primer sets 515F (5′-GTGCCAGCMGCCGCGG-3′) and 907R (5′-CCGTCAATTCMTTTRAGTTT-3′; Gao et al., 2015). The primers ITS1F (5′-CTTGGTCATTTAGAGGAAGTAA-3′) and ITS2R (5′-GCTGCGTTCTTCATCGATGC-3′) were used to amplify fungal ITS genes (Orgiazzi et al., 2012). A qPCR was performed in a TransGen AP221-02 reaction system containing 4 μL (2×) FastPfu Buffer, 0.8 μL Primer (5 μM), 2 μL dNTPs (2.5 mM), 0.4 μL FastPfu Polymerase, 10 ng template DNA, 0.2 μL BSA, and mixed 20 μL ultra-pure water. The purified PCR products were subjected to paired-end sequencing using a Miseq Illumina platform (Majorbio Bio-Pharm Technolggy Co., Ltd., Shanghai, China). The raw sequences were analyzed using a Trimmomatic v.0.32 (Bolger et al., 2014). We used FLASH to assemble paired-end clean reads which were merged as original tags, and the sequences with quality scores below 20 and/or lengths less than 150 bp were removed (Magoč and Salzberg, 2011). The denoised and sorted raw sequences were clustered into operational taxonomic units (OTUs) by using the UPARSE method with 97% identity threshold (Caporaso et al., 2010; Edgar, 2013). The taxonomic identities of the fungi and bacteria were assigned using RDP Classifier 2.2 (Wang et al., 2007) based on comparison with the UNITE 7.2 (Kõljalg et al., 2013) database and SILVA 128 (Quast et al., 2012), respectively. To compensate for different sequencing depths, samples were rarefied to an even depth of 69,041 reads for 16S and 45,955 for ITS sequences.

### 2.6. Data analysis

All analyses were conducted in R software (version 4.1.1). Before statistical analysis, the normality of the data was checked by a Shapior–Wilk’s test and the homogeneity of variance was test by a Levene’s test. One-way analysis of variance (ANOVA) combined with a Turkey’s HSD test were used to compare the means in test parameters between FTCs treatments. A Student’s t-test was performed to analyze the differences in abiotic and biotic properties at two soil depths (0–10 cm and 50–60 cm). Principal component analysis (PCA) was performed to evaluate changes in the DOM chemical compositions under different FTCs treatments with the ‘factoextra’ package. After calculating the Bray-Curtis distances, principal coordinates analysis (PCoA) was used to analyze the dissimilarity of soil microbial composition at the OTU level. Differences in the microbial communities between FTC treatments were further tested by analysis of similarities (ANOSIM) and non-parametric multivariate analysis (ADONIS) using the ‘vegan’ package. Mantel tests were implemented to interpret the significance of soil properties on the microbial compositions. The relationships between response variables and explanatory variables under FTCs treatments were tested using ordinary least squares (OLS). Co-occurrence networks were inferred for each soil layer (9 samples per layer) between the bacteria OTUs (abundance >0.05%) and DOM molecules based on the Spearman correlation matrix using the ‘psych’ package. To reduce network complexity, only individual DOM molecules with their abundance >0.01%, a correlation coefficient |*R*| > 0.8, and *p* < 0.001 were retained for further analysis. The co-occurrence networks were visualized by the Cytoscape 3.9.0 software. Redundancy analysis (RDA), following a Monte Carlo permutation test (999 permutations) was conducted to evaluate the influence of soil chemical parameters on bacterial community composition under FTCs treatment. The soil chemical parameters were first examined to reduce the collinearity by eliminating predictors with VIF > 10. A forward selection procedure on environmental variables were performed by the ‘ordiR2step’ function in the vegan package to select the descriptors that affect bacterial community composition. Forward selection is a type of stepwise regression that starts with an empty model and adds variables one by one. In each forward step, it adds the one variable that gives the single best improvement to the regression model and this process is carried out by an automatic procedure.

## 3. Results

### 3.1. Soil chemical properties

**Figure 1:**
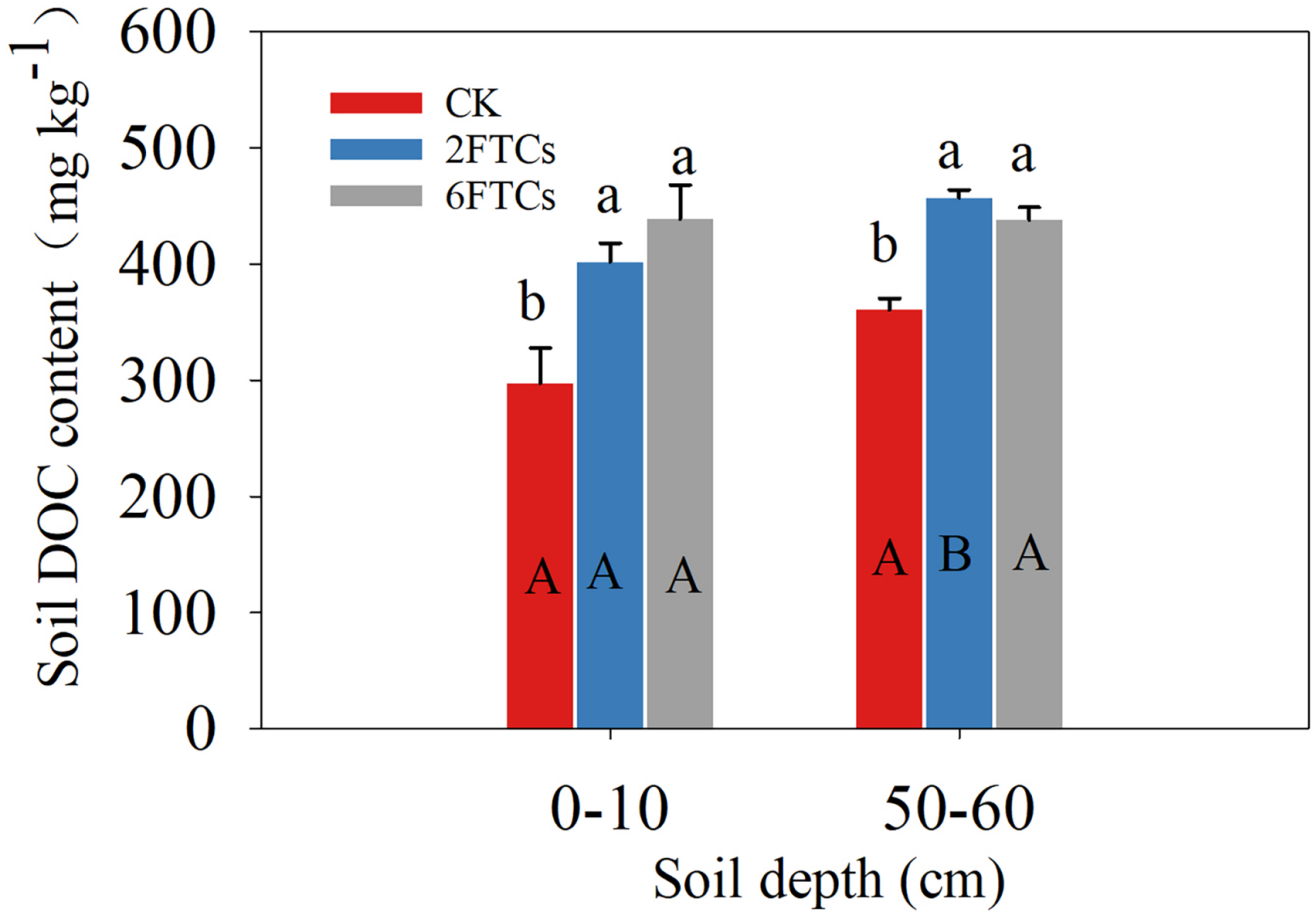
DOC contents in the 0–10 cm and 50–60 cm soil layer under different freeze–thaw cycles. CK was cultured at 5°C, while 2FTC, and 6FTC represent freeze–thaw treatment of 2 cycles and 6 cycles, respectively. Different lowercase letters indicate significant differences within each freeze–thaw treatment (*p* < 0.05). Different capital letters showed significant differences between the two soil dephts (*p* < 0.05).

Soil TC, TN, C/N, pH and NH_4_^+^-N were not significantly different among the FTCs treatments. However, FTCs significantly decreased the subsurface soil NO_3_^—^N content (Table 1). FTCs significantly increased the soil DOC content in the surface and subsurface layers from 35.04 to 47.57% and from 26.73 to 21.44%, respectively (Figure 1).

### 3.2. DOM chemical properties

**Figure 2:**
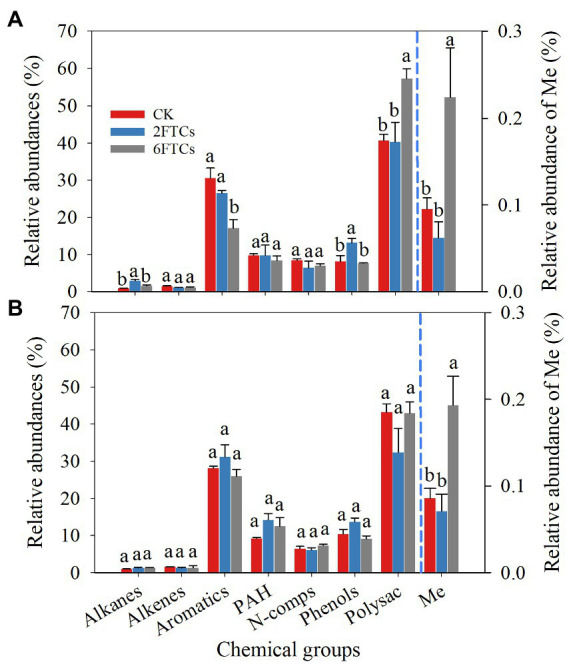
Relative abundances of 8 chemicals groups of DOM in different freeze–thaw cycles. **(A)** 0–10 cm; **(B)** 50–60 cm. PAH, Polyaromatics; N-comps, N-compounds; Polysac, Polysaccharide compounds; Me, Fatty acid methy esters. CK, 2FTC, and 6FTC represent cultured at 5°C, freeze–thaw treatment of 2 cycles and 6 cycles, respectively. The relative abundances of Me are represented on the right axis. Different lowercase letters indicate significant differences within each freeze–thaw treatment (*p* < 0.05).

A total of 121 pyrolytic compounds were identified from DOM extracts (Table S1). The pyrolytic compounds were grouped according to their chemical similarity and probable origin in the following categories: alkanes, alkenes, aromatics, polyaromatics (PAH), nitrogen compounds (N-comps), phenols, polysaccharides (Polysac), and fatty acid methyl esters (Me; Figure 2). In both surface (0–10 cm) and subsuface soils (50–60 cm), the relative abundance of polysaccharides (41.94%) was the highest, followed by that of aromatics (29.32%). The relative abundances of PAH, N-comps, and phenols were less than 10%. The relative abundances of alkanes and alkenes were less than 1.6% (Figure 2). The relative abundance of Me was two orders of magnitude lower than that of other compounds (Figure 2). In topsoil, the 6FTCs treatment significantly increased the relative abundance of polysaccharides by 40.76%, and increased the relative abundance of Me by 1.35-folds (Figure 2A). Similarly, the 2FTCs treatment significantly increased the relative abundance of alkanes by 2.32-folds and increased the relative abundance of phenols by 61.94% (Figure 2A). On the contrary, the relative abundance of aromatics significantly decreased from 30.53 to 17.04% under the 6FTCs treatment (Figure 2A). In the deeper soil layer, only Me showed a significant difference under FTCs treatments (Figure 2B), i.e., increased by 2.25-folds under the 6FTCs treatment.

**Figure 3:**
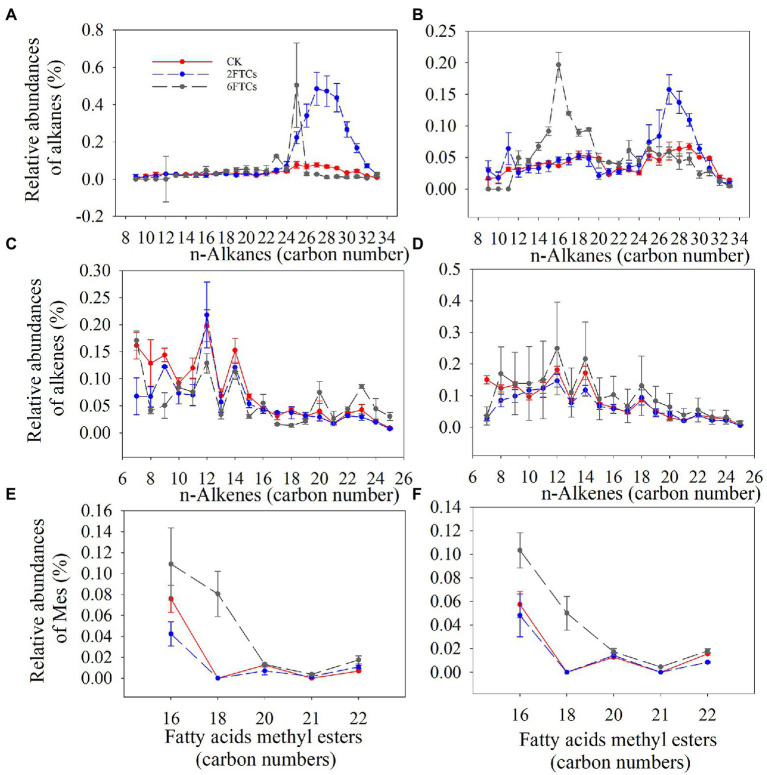
Distribution and relative abundance of Alkane in **(A)** 0–10 cm; **(B)** 50–60 cm. Alkene in **(C)** 0–10 cm; **(D)** 50–60 cm. Fatty acid methyl esters in **(E)** 0–10 cm; **(F)** 50–60 cm. CK, 2FTCs, and 6FTCs represent culture at 5°C, two freeze–thaw cycles, and six freeze–thaw cycles, respectively.

In the topsoil, 2FTCs significantly increased the relative abundance of long-chain alkanes (C25-C32), while 6FTCs only significantly increased C23 and C25 (Figure 3A). In the deeper soil, 2FTCs tended to significantly increase long-chain alkanes (C26–C30), but 6FTCs significantly increased short-chain alkanes (C13–C19; Figure 3B). The n-alkane series exhibited even-to-odd predominance, which was not affected by FTCs treatments (Figures 3C, D). A homolog series of n-fatty acids (C16–C22) was found in all DOM extracts samples with a maximum at C16 (Figures 3E, F). C18 was increased significantly in surface soil under 6FTCs treatment, and C16 and C18 also increased considerably in deep soil (Figures 3E, F).

The PCA of DOM chemical components for each soil layer is presented in Supplementary Figure S2. The first two axes of the PCA explained 37.16 and 22.37% of the overall variation for the topsoil (Supplementary Figure S2a). For deep soils, the first two axes explained 65.76% of the variation (PC1 = 44.53%; PC2 = 21.23%) in DOM chemical composition (Supplementary Figure S2c). PCA sample scores showed that 6FTCs had a greater impact on DOM chemical compositions in both surface and deep soils (Supplementary Figures S2b, d) than 2FTCs.

### 3.3. Microbial community structure and diversity

**Figure 4:**
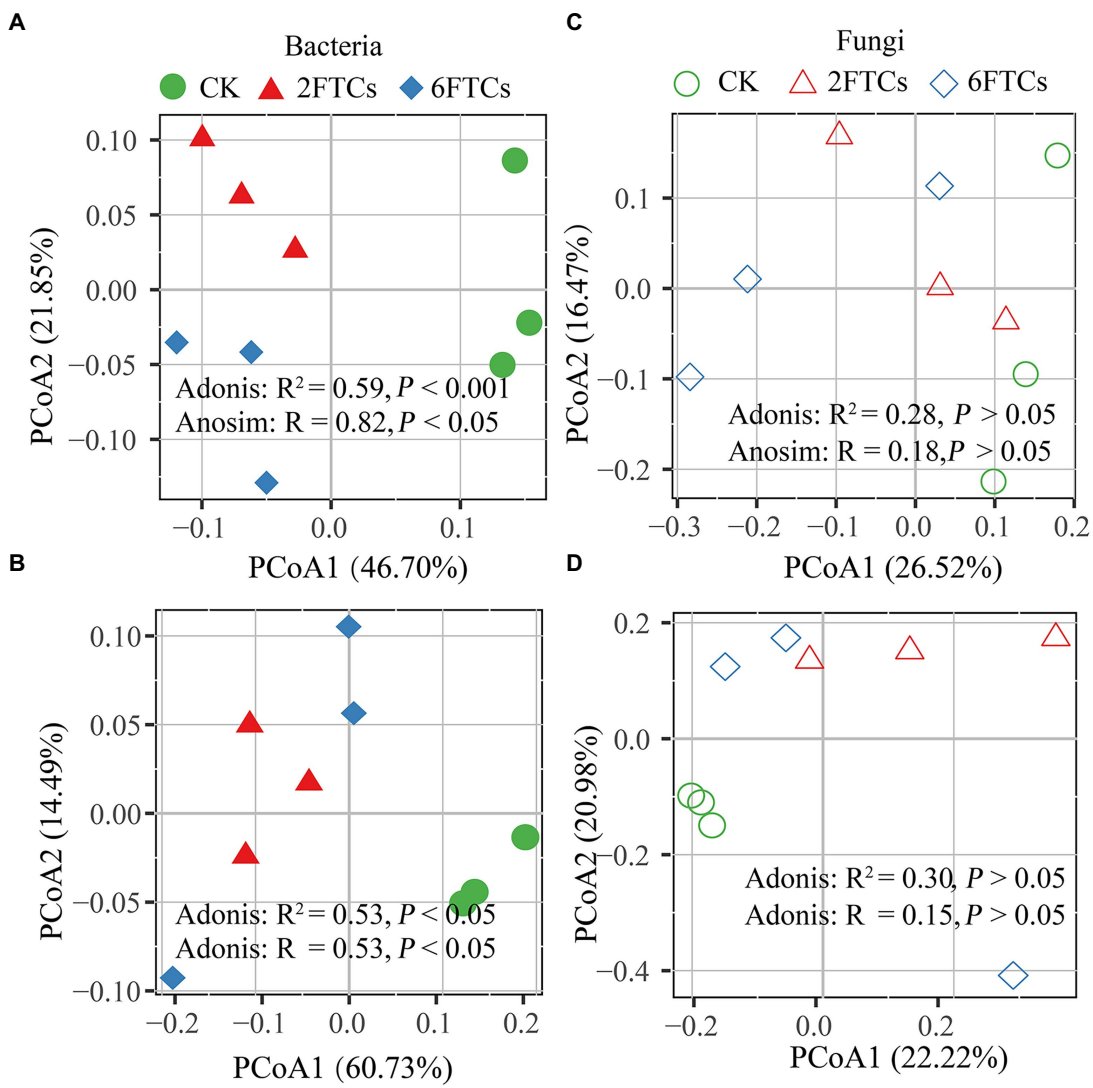
PCoA analysis of the bacterial community composition in **(A)** 0–10 cm; **(B)** 50–60 cm, and the fungal community composition in **(C)** 0–10 cm; **(D)** 50–60 cm based on the Bray-Curtis distance in different freeze–thaw treatments. CK, 2FTCs, and 6FTCs represent culture at 5°C, two freeze–thaw cycles, and six freeze–thaw cycles, respectively.

**Figure 5:**
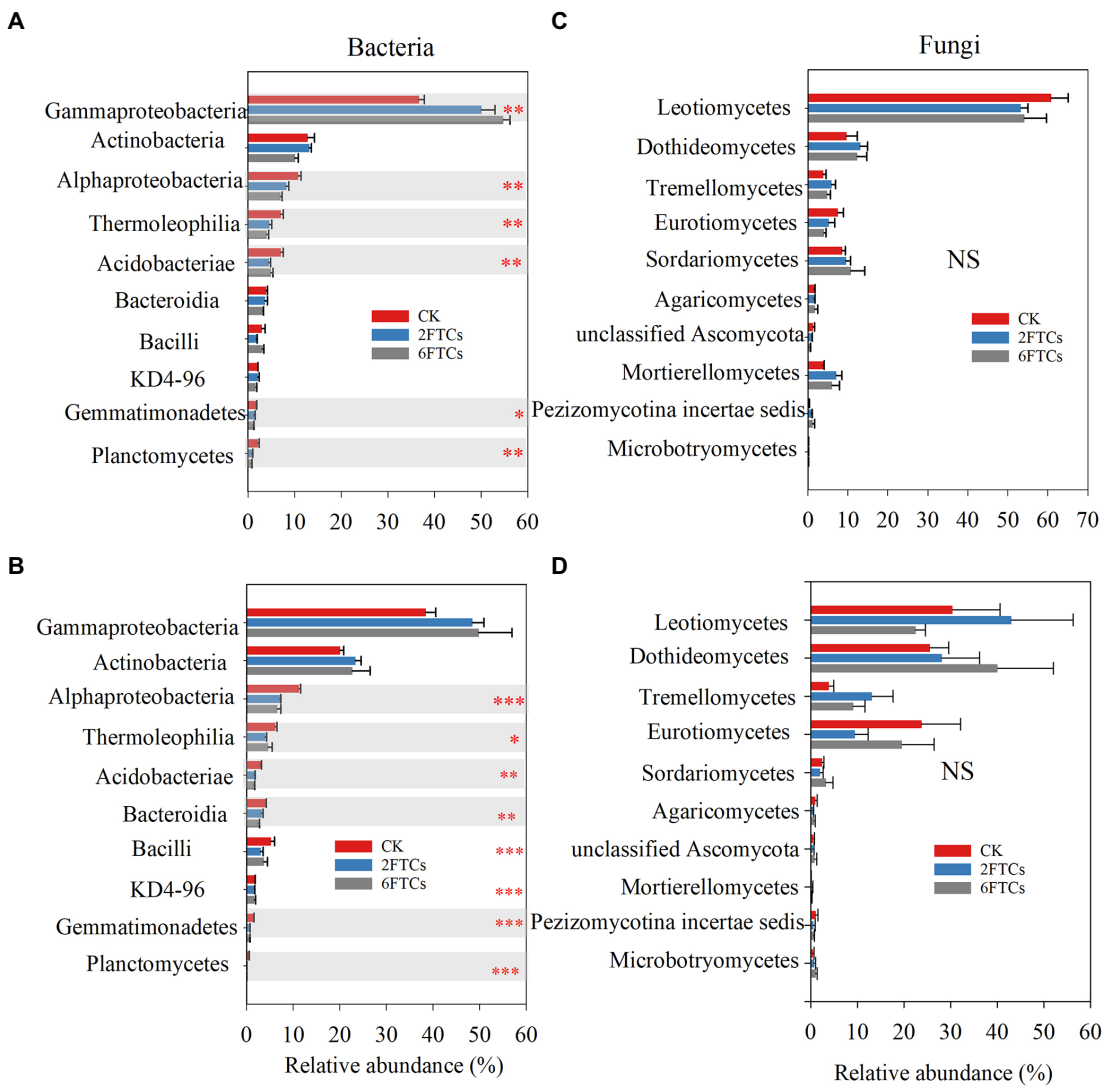
The relative abundance of dominant bacterial class in **(A)** 0–10 cm; **(B)** 50–60 cm, and fungal class in **(C)** 0–10 cm; **(D)** 50–60 cm in response to different freeze–thaw cycles. CK, 2FTCs, and 6FTCs represent culture at 5°C, two freeze–thaw cycles, and six freeze–thaw cycles, respectively. *, **, *** indicate significant differences at *p* < 0.05, < 0.01, and < 0.001, respectively. NS means no significant difference between the two soil depths.

PCoA analysis indicated that FTCs had significant effects on bacterial community but not fungal community in the two soil layers (Figure 4). The dominant phyla of bacteria were *Proteobacteria* (48.60%) and *Actinobancteria* (24.55%; Supplementary Figures S3a, b). At class level, *Gemmaproteobacteria*, *Actinobacteria, and Alphaproteobacteria,* accounted for 71.89% of the total bacterial abundance (Figures 5A, B). In the topsoil, FTCs significantly increased the relative abundance of *Gemmaproteobacteria*, but clearly decreased the relative abundances of *Alphaproteobacteria*, *Thermoleophilia*, *Acidobacteriae*, *Gemmatimonadetes*, and *Planctomycetes* (Figure 5A). Similarly, in addition to the decreased bacterial classes listed above, FTCs also significantly reduced the relative abundances of *Bacilli* and *KD4-96* in deep soil (Figure 5B). FTCs significantly changed the Shannon index of bacterial alpha diversity regardless of the soil layer (Supplementary Figure S4). For soil fungi community, the dominant fungal phyla in the control soils were *Ascomycota* (87.01%) and *Basidiomycota* (5.55%; Supplementary Figures S3c, d), and the dominant classes were *Leotiomycetes* (45.66%), followed by *Dothideomycetes* (17.58%; Figures 5C, D). Fungal community composition did not respond sensitively to FTCs, either at the phylum or class level (Figures 5C, D,  Supplementary Figures S3c, d). Furthermore, the Shannon index of fungi did not change significantly under FTCs treatment (Supplementary Figure S4).

### 3.4. Association between soil chemical properties and microbial community

**Figure 6:**
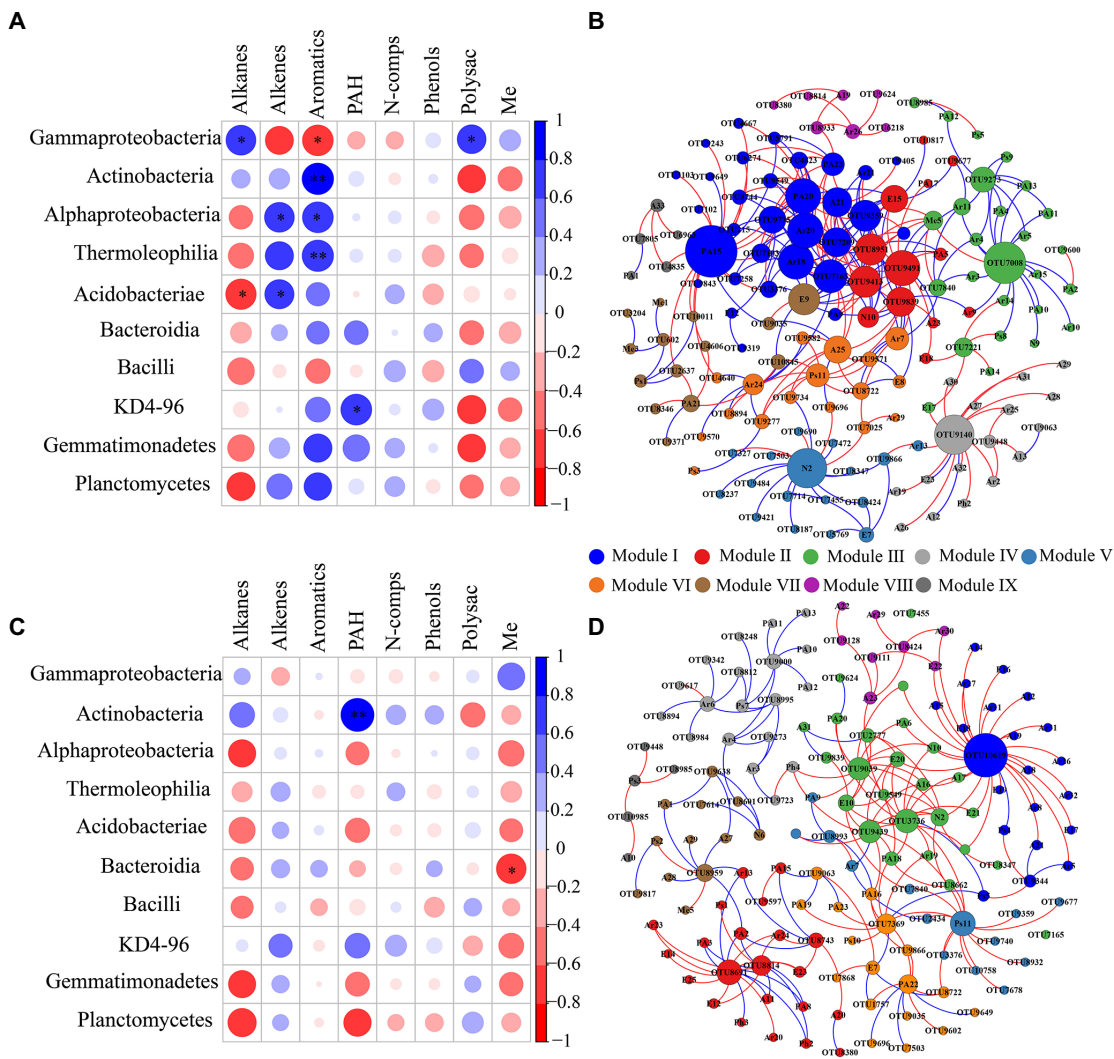
Spearman correlation analysis of the relative abundance of DOM chemical composition with the relative abundance of dominant bacterial class in **(A)** 0–10 cm; **(C)** 50–60 cm; *, **, *** indicate significant differences at *p*< 0.05, < 0.01, and < 0.001, respectively. The occurrence network between total DOM molecules and microbial OTUs based on Spearman correlation (|*r*| > 0.8, *p* < 0.001) in **(B)** 0–10 cm; **(D)** 50–60 cm. Positive and negative correlations are represented in blue and red lines, respectively. The size of each node is proportional to the number of connections. The color of the node indicates the module of the cluster. Abbreviations for DOM molecules are as in Supplementary Table S1.

**Figure 7:**
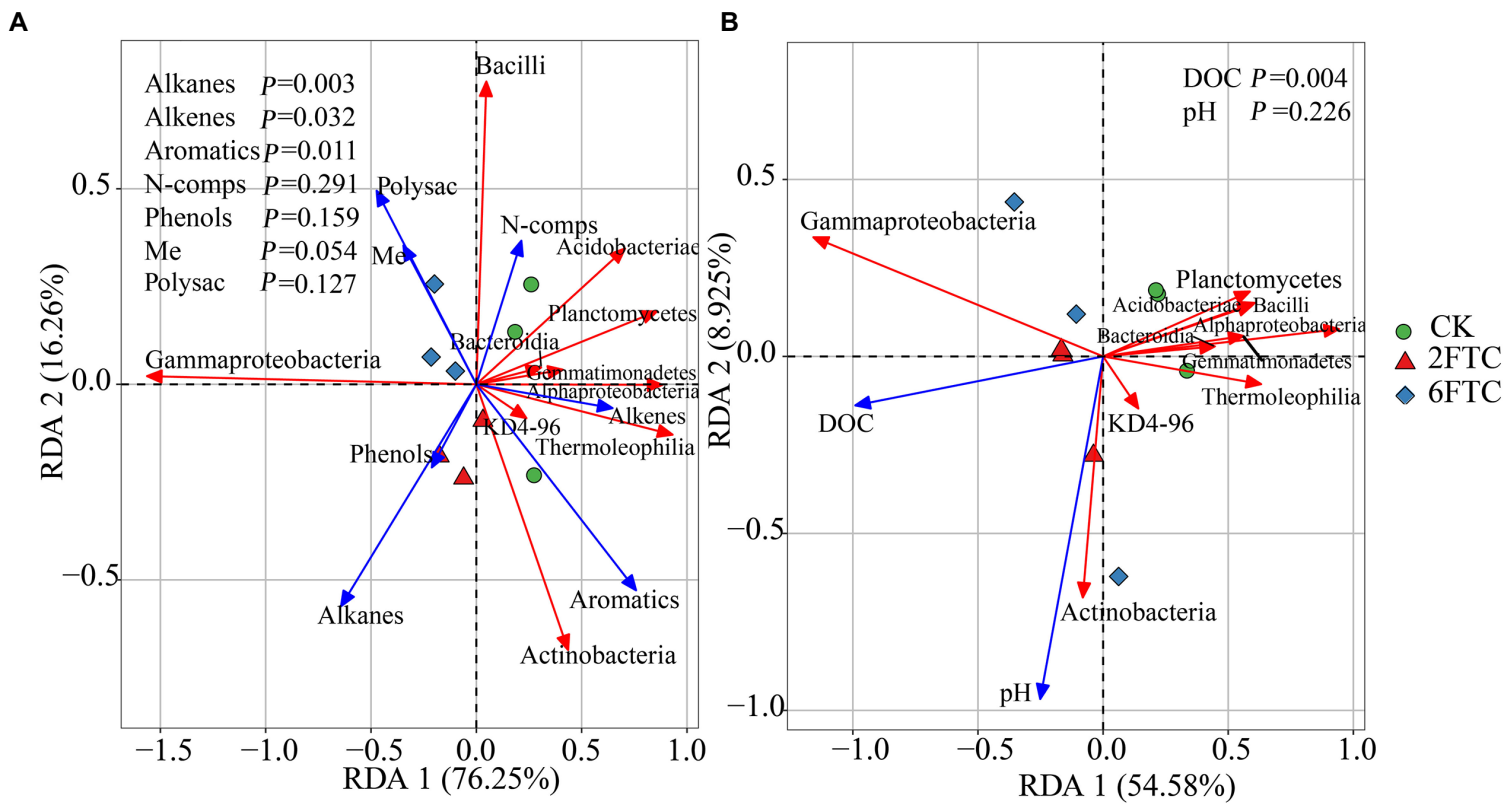
Redundancy analysis (RDA) of soil bacterial community composition at the class level as a function of soil chemical properties in **(A)** 0–10 cm and **(B)** 50–60 cm. Blue vectors represent trajectories of soil chemical properties; Red vectors represent dominant bacterial classes. Soil chemical parameters with significant effects on major bacterial classes were assessed at the 0.05 level (Monte Carlo tests with 999 permutations).

**Figure 8:**
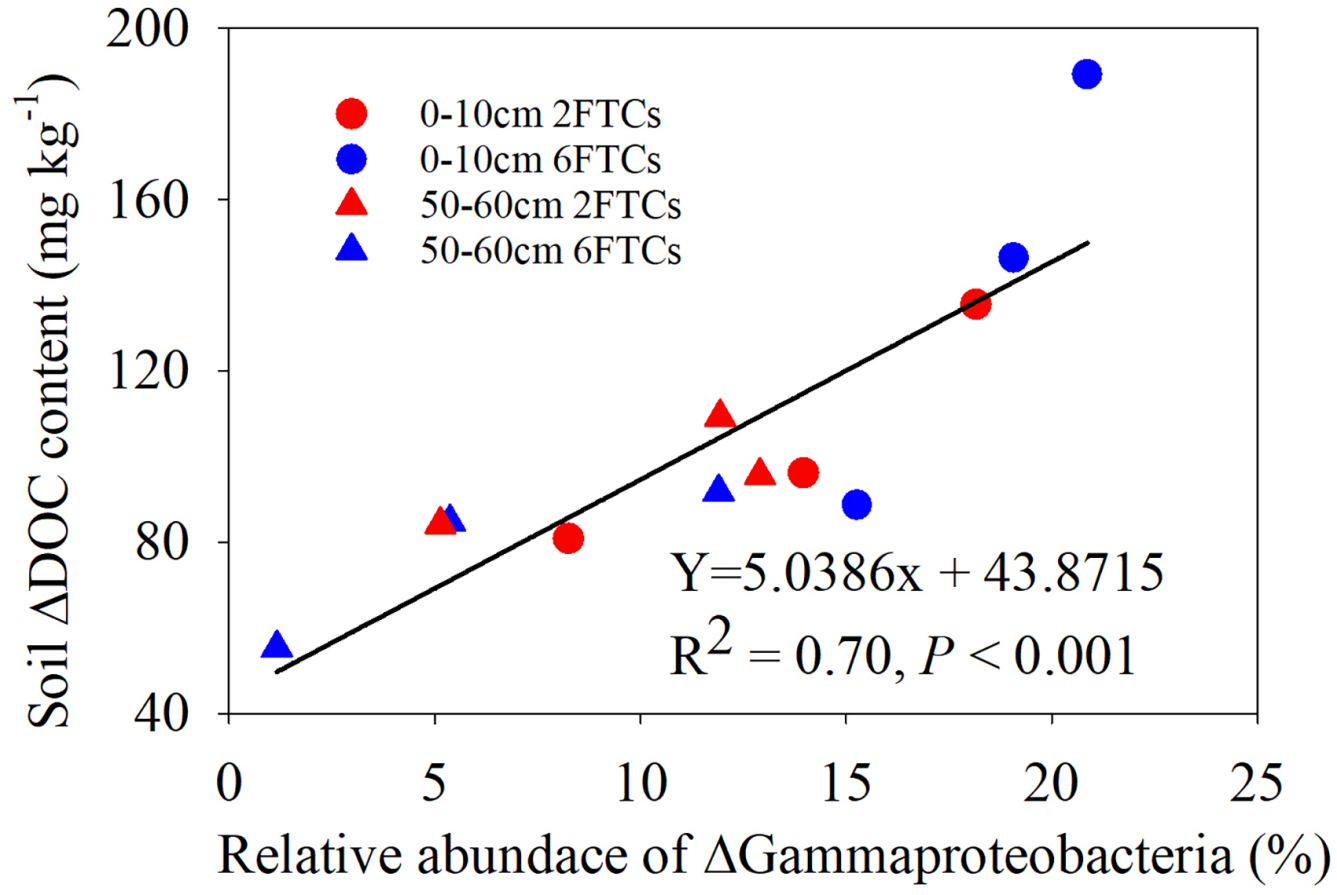
The bivariate relationship between relative abundance of ⌂*Gammaproteobacteria* and soil ⌂DOC contents in freeze–thaw cycles.

In the topsoil, aromatic compounds were positively correlated with *Actinobacteria*, *Alphaproteobacteria* and *Thermoleophilia,* but were negatively correlated with *Gammaproteobacteria.* Alkenes were positively associated with *Alphaproteobacteria* and *Acidobacteriae*, whereas Alkanes showed a negative correlation with *Acidobacteriae*. At OTU level, OTU7008, which belonged to members of *KD4-96, Chloroflexi*, showed strong positive (blue lines) correlation with recalcitrant compounds (Aromatics and PAH; Figure 6B). OTU9140 affiliated to members of *Solibacterales*, *Acidobacteriae*, and was significantly negatively correlated with long-chain alkanes (A26–A32; Figure 6B). Pyridine (N2), as a small nitrogen-containing compound, showed a significant positive association with *Acidobacteriae* (OTU8237, OTU9421, OTU8187, OTU7455, and OTU7472; Figure 6B). A Mantel test revealed that soil DOC, alkenes and aromatics were the dominant factors which independently affected the bacterial community structure (Table S2). In deep soil, *Bacteroidia* were negatively related to Me. *Actinobacteria* had a positive correlation with PAH (Figure 6C). Specifically, OTU9000 affiliated to *Actinobacteria*, was significantly positively correlated with PAH (Figure 6D). OTU10619, belonging to *Gammaproteobacteria*, was a keystone member with the highest degree of connectivity. It was significantly negatively correlated with short-chain alkanes and aromatics (Figure 6D). A Mantel test further indicated that DOC and Me were the most important factors affecting the bacterial community composition (Supplementary Table S2). RDA analysis revealed that DOM chemical compounds explained 92.51% of the bacterial community changes in the topsoil among FTCs treatments (Figure 7A). But in deep soil the DOC quantity, combined with soil pH, explained 63.50% of bacterial community variation (Figure 7B). Furthermore, bivariate regression showed that DOC increments were significantly positively correlated with *Gammaproteobacteria* increments under FTCs treatment (Figure 8).

## 4. Discussion

### 4.1. The effects of FTCs on soil dissolved C and N concentrations

FTCs notably increased soil DOC contents in the top and deep soil layers. Increased DOC contents are largely attributed to disruption of soil aggregates, lysis of microbial cells, and the reduction of microbial immobilization capacity (Gao et al., 2021). It was reported that about half of the microbial populations died when the temperature drops below −10°C during the first FTCs (Sawicka et al., 2010). That lysis of soil microbial cells directly leads to the spillover of micro-molecules such as phosphate, amino acids, and polysaccharides (Larsen et al., 2002; Meisner et al., 2021). Also, repeated expansion and contraction of soil aggregates causes disintegration of soil macroaggregates, resulting in the release of dissolved C and N (Gao et al., 2018). Furthermore, the decreased of immobilization ability of microorganisms to unstable substrates in FTCs environments indirectly led to DOC increase. Surviving soil microorganisms may utilize these active substrates triggered by FTCs, thereby enhancing soil C mineralization ability (Nielsen et al., 2001). The significant reduction in soil NO_3_^−^-N found in deep soils can be related to the finding that soil denitrifying bacteria are more tolerant to freezing temperatures than nitrobacteria (Smith et al., 2010). Denitrification recovers rapidly once the soil starts to thaw, resulting in NO_3_^−^-N consumption (Müller et al., 2003).

### 4.2. Effects of FTCs on the chemical composition of DOM

FTCs significantly decreased the relative abundance of aromatic compounds, but increased the relative abundance of polysaccharides, phenols and Me in the topsoil layer. FTCs thus tended to increase the biodegradability of DOM, which confirmed the first hypothesis. Indeed, the hydrophilic neutral fraction of DOM, represented by high contents of organic acids, carbohydrates, and proteins typically enhances biodegradability. In contrast, hydrophobic and aromatic structures reduce DOM biodegradability possibly due to their recalcitrance or inhibition of enzymatic activity (Marschner and Kalbitz, 2003).

The alkanes (A8–A33) and alkenes (E7–E25) made up only a small fraction of the pyrolysis products. Long-chain (A25–A33) alkanes and alkenes are typically derived from microbial lipids and plant biopolymers (e.g., cutin, suberin), while mid-chain and short-chain aliphatics can be attribute to microbial polymers (Buurman et al., 2007a; Vancampenhout et al., 2010). The latter may also represent parts of longer chains, that were degraded by microorganisms (chain-length shortening; Buurman et al., 2007b; Yassir and Buurman, 2012). So the release of long-chain alkanes by FTCs in our experiment may be attributed to the rupture of microbial cell membranes, while the variation of short chains might be attributed to variation in microbial decomposition. Fatty acid methyl esters (Me) can be generated by the cyclization of fatty acids (FAs) and phenolic hydroxyl groups (Musadji and Geffroy-Rodier, 2020). Generally, long-chain FAs ascribe to microbial degradation or microbial lipid fragments (Sousa et al., 2007), and FAs are typically combined with long-chain fatty alcohols or sterols to form esters (Chiavari et al., 1994). Me dominated by C16 and C18, are indicative for autochthonous (bacterial) origin (Kaal et al., 2017). Therefore, the increase in Me abundance particularly C16 and C18 caused by FTCs in our experiment could be related to microbial degradation or the accumulation of microbial lipid biomacromolecules (Figure 3). The interaction of aliphatic compounds and microorganisms is detailed in Section 4.3.

Aromatic compounds formed during the pyrolysis process mainly originate from proteins, lignin, carbohydrates and charcoal (Yassir and Buurman, 2012). Benzene (Ar1) and toluene (Ar2) were the most abundant products in our pyrograms (≈21% of total relative abundance). The potential source of benzene is mainly condensed aromatic structures, while possible sources of toluene and other alkylbenzenes (Ar19–Ar32) are proteins, tyrosine-containing peptide, lignin, and polysaccharides (De la Rosa et al., 2012). The increase of benzene content in SOM may be a caused by accelerated aliphatic cyclization or humification of organic matter (Ayuso et al., 1996). Thus, the decrease of benzene content in DOM extracts under FTCs treatment may be due to the reduced DOM humification, suggesting that the DOC released by FTCs was more conducive to microbial mining rather than C sequestration.

Some pyrolytic compounds were phenol and alkyl phenols, which can be derived from any phenolic precursor such as tannin, lignin, proteinaceous biomass, and carbohydrates (Vancampenhout et al., 2009). However, since phenols are less fractions of the pyrograms of polycarboxylic acids and proteins, the high proportion of phenols in DOM pyrolysis products may be related to degraded lignin (Rombolà et al., 2022). High methoxyphenol contents have been reported in DOM, which were considered to be the degradation products of lignin-derived DOM (Neff et al., 2006). Degradation of lignin is an important factor controlling DOM production in litter decomposition (Kalbitz et al., 2006). So we suggest that the increase in phenol yields by FTCs may be related to lignin degradation.

FTCs increased the relative proportion of polysaccharides in the topsoil layer. Polysaccharides that can be derived from microorganisms include benzofuran, furfural, and methylfuran while residuals from plant material include levoglucosan and levomannosan (Verde et al., 2008; Vancampenhout et al., 2010). Only microbial derived polysaccharide compounds were found in our study. These compounds are characterized by long residence times due to their recycling in SOM decomposition and humification (Gleixner et al., 2002).

N-compounds identified upon pyrolysis in soils generally originate from peptides, amino acids, and proteins. Pyridine (N2) and its derivatives (N6) can be formed by microbial degradation of plant lignin and other phenolic substances under NH_3_^_^enriched conditions (Buurman et al., 2007b). Because of the ubiquity of peptides and amino acids, N-compounds cannot be specifically attributed to plant and microbial sources, with the exception of chitin (De la Rosa et al., 2012). Peptides tend to adsorb on the surface of soil minerals, thereby improving their stability (Lützow Lützow et al., 2006). PAH are generally considered to be products of charred materials (e.g., charcoal), or the result of cyclization reactions during pyrolysis (Rumpel et al., 2007; Rombolà et al., 2022). However, PAH represented by methylnaphthalene (PA8) and phenanthrene (PA18) are produced by the cleavage of unsaturated fatty acids (Wagner et al., 2018). FTCs had no significant effect on their relative abundance, which may be related to their recalcitrance.

### 4.3. Association between DOM composition and bacterial community

To date, several researches have aimed to elucidate the link between DOM chemical diversity and bacterial community diversity (Underwood et al., 2019; Ling et al., 2022). The interactions between them can be bidirectional. Specifically, (i) DOM chemical composition has been shown to drive the composition of the microbial community, and in general more complex DOM molecules correspond to relatively high microbial abundance and diversity (Li et al., 2018). It has been demonstrated that a diversity of DOM molecules trigger an increase in bacterial diversity, especially the abundance of *Betaproteobacteria*, *Gammaproteobacteria* and *Flavobacteria* has been related to complex DOM profiles (Zhao et al., 2019) (ii) Specific microbial communities moreover have been reported to exclusively decompose specific DOM substrates (Ling et al., 2022). e.g., *Nitrospira* was negatively associated with DOM recalcitrant components, revealing that *Nitrospira* specialized in decomposing this type of DOM components (Li et al., 2018). The latter can be interpreted by the affinities of soil microorganisms for individual organic matter molecules. Considering the utilization of DOM by soil microorganisms, higher microbial biomass resulted in less C substrates remaining after consumption, especially in closed systems without substrate replenishment. A negative correlation has been used in the literature to represent the affinity of microorganisms for C compounds in a closed system without continuous substrates supplied (Ling et al., 2022). Although the FTCs treatment lead to a continuous release of DOM, which can be considered equivalent to providing a semi-continuous substrates for microorganisms, we propose that a positive correlation between soil microorganisms and DOM molecules in our experiments also indicates that microorganisms preferentially utilize individual DOM compounds.

The phylum *Proteobacteria* have been identified as *copiotrophic* groups (r-strategy), which are not only significantly positively correlated with labile DOM components (particularly polysaccharide compounds), but also with recalcitrant DOM compounds (Ho et al., 2017; Li et al., 2019). *Gammaproteobacteria* have been reported to play a crucial role in the degradation of alkanes and labile carbohydrates under aerobic conditions (Martirani-Von Abercron et al., 2016). This contradiction needs detailed taxonomy at lower phylogenetic levels. For example, *Gammaprotepbacteria* (i.e., *Pseudomonadales*, *Enterobacteriales*) and *Betaproteobacteria* (i.e., *Burkholderiales*) responded quickly to unstable C (Di Lonardo et al., 2017), whereas the class of *Alphaproteobacteria* (i.e., *Sphingomonadalaes*) is inclined to utilize both labile sucrose and recalcitrant compounds (Goldfarb et al., 2011). *Microvirga* (*Alphaproteobacteria*) aggregated in heavy metal contaminated and nutrient-deficient soils (Igwe and Vannette, 2019). Thus, the changes in the corresponding C components associated with *Proteobacteria* suggested that this group can adapt to C substrates with diverse chemical recalcitrance (Goldfarb et al., 2011). It was also confirmed by the results of the utilization of different DOM components by *Gammaproteobacteria* and *Alphaprobacteria* in our study. In addition, *Gammaproteobacteria* were observed to have a strong positive correlation with DOC increment under FTCs treatment (Figure 8). The release of active components (e.g., DOC, DON) after each freeze–thaw is comparable to a repeated addition of multiple substrates in soil priming experiments. We proposed that repeated multiple substrates release under FTCs treatments may increase the living microbial activity and SOM mineralization rate when the substrate input of each repeated addition exceeds the threshold amount required for soil priming effects (Fontaine et al., 2003).

The phylum *Actinobacteria*, which is a representative of *oligotrophic* bacteria (K- strategy), could grow slowly in low-nutrient soils and tolerate harsh conditions. The relative proportion of *Actinobacteria* did not change significantly after FTCs treatment. Classes of *Actinobacteria* and *Thermoleophilia* both belonged to the phylum *Actinobacteria*, and *Actinobacteria* were not affected by freeze–thaw, while the relative abundance of *Thermoleophilia* was significantly reduced under FTC treatment. One possible reason is that *Thermoleophilia* are known to be moderately thermophilic and oil-loving (Foesel et al., 2016). Recent studies have indicated *Actinobacteria* and *Thermoleophilia* correlated to both unstable components (particularly carbohydrates) and recalcitrant compounds *via* co-metabolism (Ling et al., 2022). Our findings only support the idea that these two classes could decompose aromatic DOM compounds. *Actinobacteria* are vital saprophytes capable of using a range of enzymes (protease, xylanase, β-glucosidase, celluases, and other ligninolytic enzymes) to decompose rhizodeposits and litter (Kabuyah et al., 2012; Manivasagan et al., 2013). These enzymes can act on amino sugars, polysaccharides, cellulose, and lignin, so that both small molecules and complex substances can be degraded (Lladó et al., 2016; Zhang et al., 2017). Moreover, the hyphal-like morphology of *Actinobacteria* facilitate their contact with organic matter and promote C mineralization. *Actinobacteria* are considered as one of the key contributors to SOC mineralization in biochar-amended soils (Jeewani et al., 2020).

Several *oligotrophic* species in the phylum *Acidobacteria* function to decompose relatively stable and recalcitrant SOM (Hale et al., 2019). *Acidobacteriae* were observed to be negatively associated with alkanes. Lipids, including alkanes and alkenes, represent a varied group of amphiphilic and hydrophobic biomolecules whose physicochemical attributes allow them to present diverse cellular functions. They can be act as components of cell membranes and membrane proteins, and can also be active intercellular and intracellular signaling molecules in energy homeostasis. Under FTCs treatment, the relative abundance of *Acidobacteriae* significantly decreased but the relative proportion of long-chain alkanes increased remarkably. Therefore, we suggest that the release of long-chain alkanes may be related to the lysis of cell membranes of *Acidobacteriae*. This was also confirmed by the results of OTU9140 (affiliated to *Solibacterales*, *Acidobacteria*) and long-chain alkanes (A26-A32) in the co-occurrence network (Figure 6B). *Acidobacteriae* were closely associated with alkenes, particularly the short-chain alkenes (E9, E12; Figures 6A, B). Short-chain aliphatic compounds generally originate from microbial polymers or from microbial degradation of longer chains (Yassir and Buurman, 2012; Nam et al., 2021). Thus, *Acidobacteriae* may play a role in degrading alkenes. *Bacilli* (*Firmicutes*), KD4-96 (*Chloroflexi*), *Gemmatimonadetes*, and *Planctomycetes* were not correlated with the DOM chemical compositions, except that KD4-96 was positively correlated with PAH in topsoil. This might be due to the reduction of microbial activity of low-abundance microorganisms under freeze–thaw environments. Another possible reason is that the DOM bioavailability has not changed significantly under the freeze–thaw treatment. Thus, in topsoil, the chemical composition of DOM is one of the major drivers of bacterial community variability under FTCs treatment, while in deeper soil, DOC content is the main factor shaping the bacterial community.

The co-occurrence network pattern reflected that taxa of the same class, or even same genus, presented diverse correlations with DOM components of distinct chemical characteristics. Specifically, there are the same associations for different categories of DOM compounds, or opposite associations for the same class of molecules. For instance, *Acidobacteriae* showed negative and positive correlations with alkanes and alkenes, respectively. *Alphaproteobacteria* presented positive correlations with alkenes and aromatic compounds. A module in the co-occurrence network corresponds to a functional cluster, indicating that microorganisms in that cluster utilize similar DOM molecules (Deng et al., 2012). OTUs in the same module may indicate that these microorganisms occupy similar ecological niches (Zhou et al., 2010). The network modules thus support the finding that specific microorganisms degrade specific substrates (Li et al., 2018).

## 5. Conclusion

This study demonstrated that freeze and thaw increased DOC contents in the surface and deep soils of a boreal forest, and changed the chemical composition of DOM. In particular, the decrease of aromatic compounds and the increase of alkanes, phenols, polysaccharides, and Me in the topsoil indicated an improvement of DOM bioavailability. Soil bacteria were more sensitive to FTCs as compared to fungi, manifesting as a decrease in relative abundance of bacterial classes (e.g., *Alphaproteobacteria*, *Thermoleophilia*, and *Acidobacteriae*) and a decrease in Shannon index. Furthermore, *Gammaproteobacteria* were dominant in freeze and thaw cycled soils and most likely induced the largest contribution to DOC release. The interactions between DOM molecules and bacterial communities showed that specific microorganisms can degrade specific substrates. In the topsoil, DOM chemical composition shaped bacterial communities, with labile C correlated to *Gammaproteobacteria*, and more recalcitrant C associated with other bacteria (e.g., *Actinobacteria*, *Alphaproteobacteria*, and *Thermoleophilia*). In comparison, DOC contents were more likely to explain the variation of bacterial communities in the deeper soil layers. This study thus provides new insights into DOC accumulation, transformation, and stability in boreal forest soils under scenarios of intensified freezing–thawing.

## Data availability statement

The datasets presented in this study can be found in online repositories. The names of the repository/repositories and accession number(s) can be found at: https://www.ncbi.nlm.nih.gov/, PRJNA899719 and PRJNA900157.

## Author contributions

YY, HF, and SC: conceptualization, methodology, data curation, and writing—review and editing. YY: software and writing—original draft preparation. YY, YG, YL, YZ, and FS: formal analysis. HF: investigation and project administration. HF and SC: resources and supervision. KV: visualization. HF and KV: funding acquisition. All authors contributed to the article and approved the submitted version.

## Funding

This research was funded by the Second Tibetan Plateau Scientific Expedition and Research Program (STEP) (No. 2019QZKK1003), the Strategic Priority Research Program of the Chinese Academy of Sciences (Nos. XDA28130100, XDA200204020, and XDA23060401), National Natural Science Foundation of China (Nos. 41977041 and 31770558), the “Thousand Talents Plan” Project of High-End Innovative Talents of Qinghai Province (No. TTPPHEITQP-2019), and Key research and development projects of Ji’an Science and Technology Bureau (20111ZDF04022). KV received an FWO sabbatical bench fee (number VWH-E1313-SAB/22/016).

## Conflict of interest

The authors declare that the research was conducted in the absence of any commercial or financial relationships that could be construed as a potential conflict of interest.

## Publisher’s note

All claims expressed in this article are solely those of the authors and do not necessarily represent those of their affiliated organizations, or those of the publisher, the editors and the reviewers. Any product that may be evaluated in this article, or claim that may be made by its manufacturer, is not guaranteed or endorsed by the publisher.

## Supplementary material

The Supplementary material for this article can be found online at: https://www.frontiersin.org/articles/10.3389/fmicb.2022.1012512/full#supplementary-material
