# Supplementary material for: Linkages between the molecular composition of dissolved organic matter and soil microbial community in a boreal forest during freeze–thaw cycles
Source: Front Microbiol. 2023 Jan 9;13:1012512. doi: 10.3389/fmicb.2022.1012512 (PMC9868181; doi:10.3389/fmicb.2022.1012512)

Supplementary information

Table S1 Quantified pyrolysis products of DOM

| Name**^†^** | Code | m/z | RT | Name | Code | m/z | RT |
| --- | --- | --- | --- | --- | --- | --- | --- |
| **n-Alkanes** |  |  |  | Pentadecylbenzene | Ar29 | 91+288 | 64.45 |
| nC9 | A9 | 57+71 | 9.08 | Hexadecylbenzene | Ar30 | 91+302 | 66.70 |
| nC10 | A10 | 57+71 | 19.22 | Heptadecylbenzene | Ar31 | 91+316 | 68.85 |
| nC11 | A11 | 57+71 | 26.15 | Octadecylbenzene | Ar32 | 91+330 | 70.91 |
| nC12 | A12 | 57+71 | 33.03 | **Polyaromatics (PAHs)** |  |  |  |
| nC13 | A13 | 57+71 | 38.31 | Naphthalene | PA1 | 128 | 31.64 |
| nC14 | A14 | 57+71 | 42.44 | 1-Methylnaphthalene | PA2 | 141+142 | 37.80,38.58 |
| nC15 | A15 | 57+71 | 45.97 | Dimethylnaphthalene | PA3 | 141+156 | 42.52 |
| nC16 | A16 | 57+71 | 49.15 | 1-Isopropylnaphthalene | PA4 | 155+170 | 46.84 |
| nC17 | A17 | 57+71 | 52.08 | 2,3,5-Trimethylnaphthalene | PA5 | 170 | 47.02 |
| nC18 | A18 | 57+71 | 54.82 | 1,2,3,4- Tetramethylnaphthalene | PA6 | 184 | 51.82 |
| nC19 | A19 | 57+71 | 57.41 | Bibenzene | PA7 | 154 | 41.54 |
| nC20 | A20 | 57+71 | 59.85 | 3-Methylbiphenyl | PA8 | 168 | 45.47 |
| nC21 | A21 | 57+71 | 62.20 | 1-Methyl-4-(4-methylbenzyl)benzene | PA9 | 181+196 | 50.2 |
| nC22 | A22 | 57+71 | 64.45 | 1,2-Dihydronaphthalene | PA10 | 115+130 | 29.29 |
| nC23 | A23 | 57+71 | 66.60 | 3-Methyl-1H-indene | PA11 | 129+130 | 29.69 |
| nC24 | A24 | 57+71 | 68.68 | 2,3-Dimethyl-1H-indene | PA12 | 129+144 | 36.44 |
| nC25 | A25 | 57+71 | 70.68 | 1,1-Dimethylindene | PA13 | 129+144 | 35.86 |
| nC26 | A26 | 57+71 | 72.58 | 1,2-Dihydro-3-methylnaphthalene | PA14 | 129+144 | 36.65 |
| nC27 | A27 | 57+71 | 74.43 | Fluorene | PA15 | 165+166 | 48.66 |
| **n-Alkenes** |  |  |  | 1-Methylfluorene | PA16 | 180 | 53.32 |
| nC7:1 | E7 | 55+69 | 4.94 | 9,9-Dimethyl-9H-fluorene | PA17 | 194 | 55.61 |
| nC8:1 | E8 | 55+69 | 7.76 | Phenanthrene | PA18 | 178 | 54.44 |
| nC9:1 | E9 | 55+69 | 12.22 | Anthracene | PA19 | 178 | 54.73 |
| nC10:1 | E10 | 55+69 | 18.64 | 3-Methylphenanthrene | PA20 | 192 | 57.46 |
| nC11:1 | E11 | 55+69 | 25.55 | 2,7-Dimethylphenanthrene | PA21 | 206 | 60.93 |
| nC12:1 | E12 | 55+69 | 32.51 | Pyrene | PA22 | 101+202 | 61.61 |
| nC13:1 | E13 | 55+69 | 37.92 | Fluoranthene | PA23 | 101+202 | 62.84 |
| nC14:1 | E14 | 55+69 | 42.13 | **N-compounds(N-comps)** |  |  |  |
| nC15:1 | E15 | 55+69 | 45.71 | 1H-Imidazole | N1 | 41+68 | 2.97 |
| nC16:1 | E16 | 55+69 | 48.91 | Pyridine | N2 | 52+79 | 6.53 |
| nC17:1 | E17 | 55+69 | 51.87 | 2-Pyrrolidinylmethanol | N3 | 70 | 8.34 |
| nC18:1 | E18 | 55+69 | 54.63 | 1-Methyl-3-vinyl-1H-pyrazole | N4 | 107+108 | 9.42 |
| nC19:1 | E19 | 55+69 | 57.44 | 1,3-Dimethylpyrazole | N5 | 95+96 | 9.65 |
| nC20:1 | E20 | 55+69 | 59.70 | 2,3-Dimethylpyridine | N6 | 106+107 | 15.09 |
| nC21:1 | E21 | 55+69 | 62.07 | Benzonitrile | N7 | 76+103 | 18.04 |
| nC22:1 | E22 | 55+69 | 64.32 | 3-Methyl-1H-Indazole | N8 | 131+132 | 25.81 |
| nC23:1 | E23 | 55+69 | 66.50 | 4-Methyl-1H-Indazole | N9 | 131+132 | 26.10 |
| nC24:1 | E24 | 55+69 | 68.57 | Tetrahyesdroisoquinoline | N10 | 104+132 | 37.19 |
| nC25:1 | E25 | 55+69 | 70.56 | Tetradecanenitrile | N11 | 43+97 | 45.62 |
| **Aromatics** |  |  |  | **Phenols** |  |  |  |
| Benzene | Ar1 | 77+78 | 4.46 | Phenol | Ph1 | 66+94 | 18.38 |
| Toluene | Ar2 | 91+92 | 6.93 | 3-Propylphenol | Ph2 | 107+136 | 23.31 |
| Ethyl-benzene | Ar3 | 91+106 | 10.81 | 3-Methylphenol | Ph3 | 107+108 | 24.93 |
| 1,3-Dimethylbenzene | Ar4 | 91+106 | 11.22 | 2-Methyl-5-isopropylphenol | Ph4 | 135+150 | 34.22 |
| Styrene | Ar5 | 78+104 | 12.33 | **Polysaccharide compounds(Polysacs)** |  |  |  |
| 1,4-Dimethylbenzene | Ar6 | 91+106 | 12.45 | Methylfuran | Ps1 | 53+82 | 3.69 |
| Propylbenzene | Ar7 | 91+120 | 16.07 | Acetic acid | Ps2 | 45+60 | 5.08 |
| 1-Methyl-2-ethylbenzene | Ar8 | 105+120 | 17.742 | 2,5-Dimethylfuran | Ps3 | 54+96 | 5.31 |
| 1,2,4-Trimethylbenzene | Ar9 | 105+120 | 18.631 | Furfural | Ps4 | 95+96 | 9.63 |
| 1,3,5-Trimethylbenzene | Ar10 | 105+120 | 20.542 | 2-Ethyl-5-methylfuran | Ps5 | 95+110 | 13.71 |
| 1,3-Dimethyl-2-ethylbenzene | Ar11 | 119+134 | 20.770 | 5-Methyl-2-furfural | Ps6 | 109+110 | 16.82 |
| 1-Propenylbenzene | Ar12 | 117+118 | 21.38 | Benzofuran | Ps7 | 89+118 | 18.78 |
| Indene | Ar13 | 115+116 | 21.97 | 2-Methylbenzofuran | Ps8 | 131+132 | 26.48 |
| Butylbenzene | Ar14 | 91+134 | 22.92 | 4,7-Dimethylbenzofuran | Ps9 | 145+146 | 33.75 |
| 1-Ethyl-3-methylbenzene | Ar15 | 91+105 | 23.61 | Dibenzofuran | Ps10 | 168 | 46.51 |
| (2-Methyl-1-propenyl)benzene | Ar16 | 117+132 | 25.25 | Oleic Acid | Ps11 | 55+97 | 52.39 |
| 1,4-Diethyl-2-methylbenzene | Ar17 | 119+133 | 27.07 | **Fatty acid methy esters (FAMEs)** |  |  |  |
| 1-Isopropenyl-4-(methoxymethyl)-Ben | Ar18 | 147+162 | 40.74 | Methyl hexadecanoate | Me1 | 74 | 58.05 |
| Amylbenzene | Ar19 | 91+148 | 30.01 | Methyl stearate | Me2 | 74 | 62.39 |
| Hexylbenzene | Ar20 | 91+162 | 36.24 | Methyl arachidate | Me3 | 74 | 67.21 |
| Heptylbenzene | Ar21 | 91+176 | 40.94 | Methyl heneicosanoate | Me4 | 74 | 69.27 |
| Octylbenzene | Ar22 | 91+190 | 44.83 | Methyl behenate | Me5 | 74 | 71.25 |
| Nonylbenzene | Ar23 | 91+204 | 48.26 |  |  |  |  |
| Decylbenzene | Ar24 | 91+218 | 51.40 |  |  |  |  |
| Undecylbenzene | Ar25 | 91+232 | 54.31 |  |  |  |  |
| Tetrapropylene-benzene | Ar26 | 91+246 | 57.04 |  |  |  |  |
| Tridecylbenzene | Ar27 | 91+260 | 59.61 |  |  |  |  |
| Tetradecylbenzen | Ar28 | 91+274 | 62.09 |  |  |  |  |

**^†^** Name, compound; Code, compound abbreviation; m/z, specific masses identified to quantification; RT, retention time.

Table S2 Mantel test results for the correlation between relative abundances of bacterial and fungal class and environmental variables at different soil depths under FTCs treatment.

| Soil variables | 0-10 cm | | | |  | 50-60 cm | | | |
| --- | --- | --- | --- | --- | --- | --- | --- | --- | --- |
|  | Bacteria | | Fungi | |  | Bacteria | | Fungi | |
|  | r | *P* | r | *P* |  | r | *P* | r | *P* |
| DOC | 0.700 | **0.002** | -0.216 | 0.817 |  | 0.445 | **0.014** | 0.183 | 0.168 |
| TC | 0.282 | 0.088 | 0.055 | 0.268 |  | -0.158 | 0.807 | -0.163 | 0.840 |
| TN | -0.209 | 0.917 | 0.265 | 0.152 |  | 0.119 | 0.164 | -0.023 | 0.495 |
| C/N | -0.150 | 0.822 | 0.013 | 0.377 |  | 0.006 | 0.412 | 0.089 | 0.339 |
| pH | 0.123 | 0.244 | 0.140 | 0.186 |  | 0.128 | 0.280 | -0.207 | 0.798 |
| NH_4_^+^-N | -0.225 | 0.990 | -0.036 | 0.522 |  | -0.109 | 0.651 | -0.242 | 0.825 |
| NO_3_^-^-N | 0.042 | 0.376 | -0.297 | 0.967 |  | 0.293 | 0.061 | 0.152 | 0.208 |
| Alkanes | 0.163 | 0.149 | -0.144 | 0.730 |  | 0.017 | 0.385 | -0.210 | 0.826 |
| Alkenes | 0.379 | **0.029** | -0.103 | 0.590 |  | -0.170 | 0.707 | -0.228 | 0.740 |
| Aromatics | 0.384 | **0.026** | -0.182 | 0.743 |  | -0.180 | 0.753 | -0.266 | 0.822 |
| PAH | -0.188 | 0.88 | -0.238 | 0.886 |  | 0.165 | 0.237 | -0.284 | 0.957 |
| N-comps | -0.189 | 0.883 | -0.079 | 0.538 |  | -0.166 | 0.817 | 0.263 | 0.083 |
| Phenols | -0.106 | 0.731 | -0.195 | 0.843 |  | 0.018 | 0.451 | -0.084 | 0.624 |
| Polysac | 0.227 | 0.107 | -0.080 | 0.617 |  | -0.096 | 0.587 | -0.194 | 0.725 |
| Me | -0.193 | 0.885 | 0.540 | 0.094 |  | 0.360 | **0.049** | 0.038 | 0.387 |

**Supplement figure captions**

**Fig. S1** (a) Air temperature variation in the study area from mid-March to mid-April in 2000 and 2020. (b) Temperature setting for indoor freeze-thaw cycle simulations. 2FTCs and 6FTCs mean two and six freeze-thaw cycles, respectively.

**Fig. S2** PCA analysis of the pyrolysis compounds of DOM in different freeze-thaw treatment. PCA loading of compounds on the first two ordination axes in (a) 0-10cm and (c) 50-60cm. PCA scores of the samples on the first two ordination axes in (b) 0-10cm and (d) 50-60cm. CK, 2FTCs, and 6FTCs represent culture at 5℃, two freeze-thaw cycles, and six freeze-thaw cycles, respectively.

**Fig. S3** The relative abundance of dominant bacteria phylum in (a) 0-10 cm; (b) 50-60 cm, and fungal phylum in (c) 0-10 cm; (d) 50-60 cm in response to different freeze-thaw cycles. CK, 2FTCs, and 6FTCs represent culture at 5℃, two freeze-thaw cycles, and six freeze-thaw cycles, respectively. *, **, *** indicate significant differences at P < 0.05, < 0.01, and < 0.001, respectively. NS means no significant difference between the two soil layers.

**Fig. S4** The Shannon index of bacteria (a) and fungi (b) in the two soil layers under different experimental treatments. CK, 2FTCs, and 6FTCs represent culture at 5℃, two freeze-thaw cycles, and six freeze-thaw cycles, respectively. Different lowercase letters indicate significant differences between experimental treatments (*P* < 0.05). Different capital letters indicate significant differences between the surface and deep soils (*P* < 0.05). NS means not significant.

**Fig. S1**


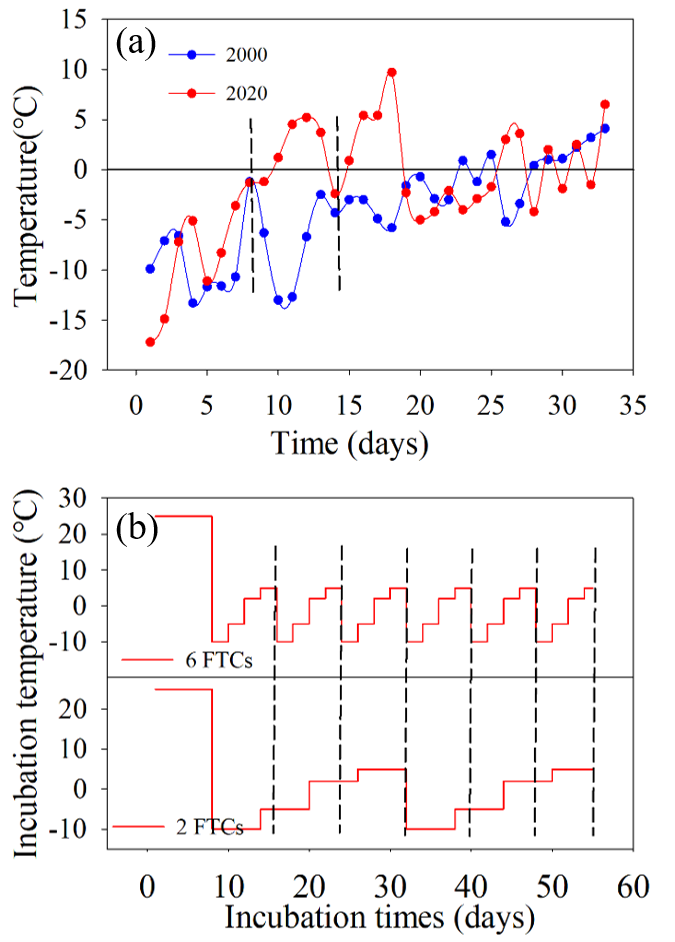


**Fig. S2**

**
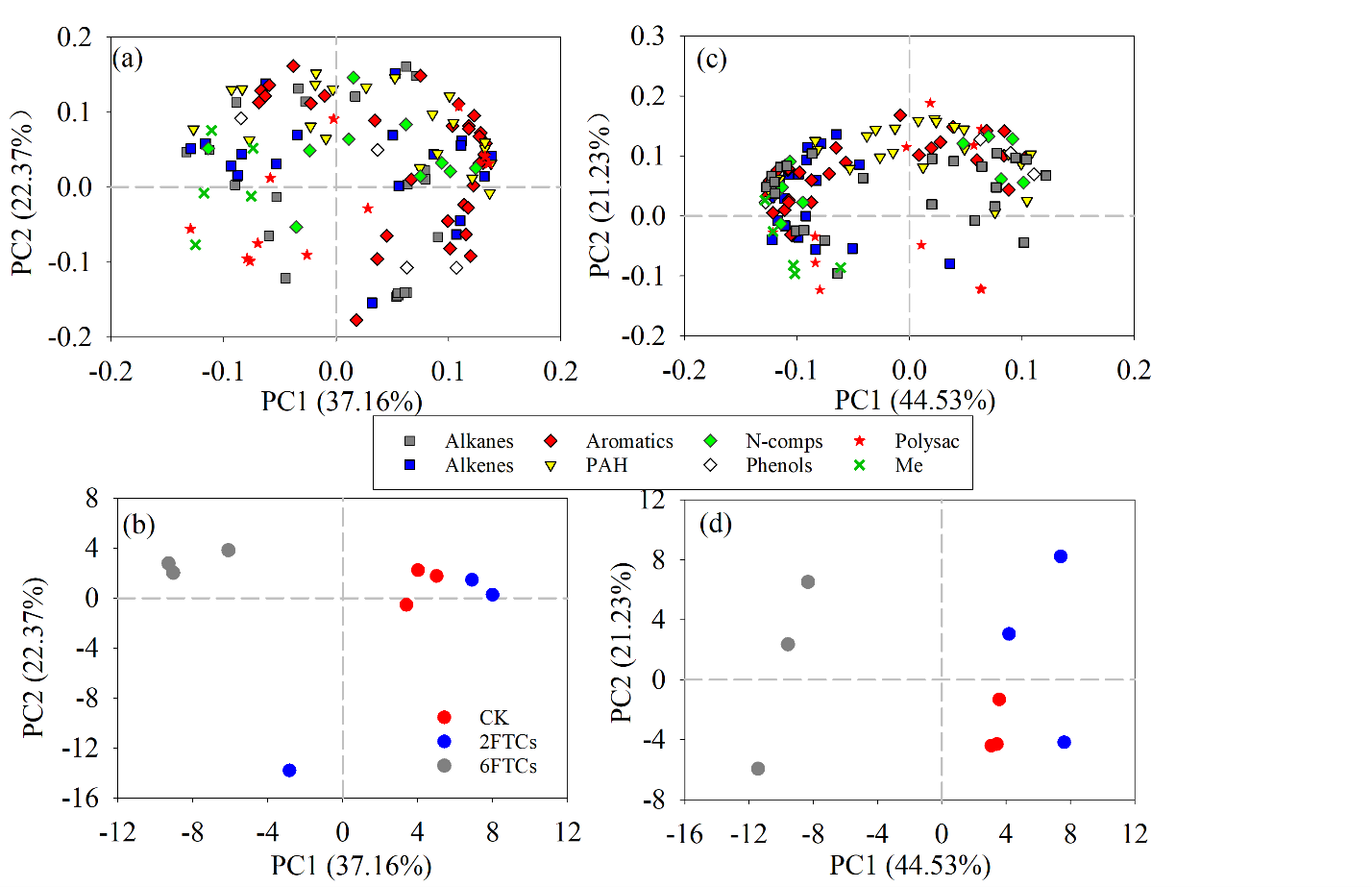
**

**Fig. S3**

**
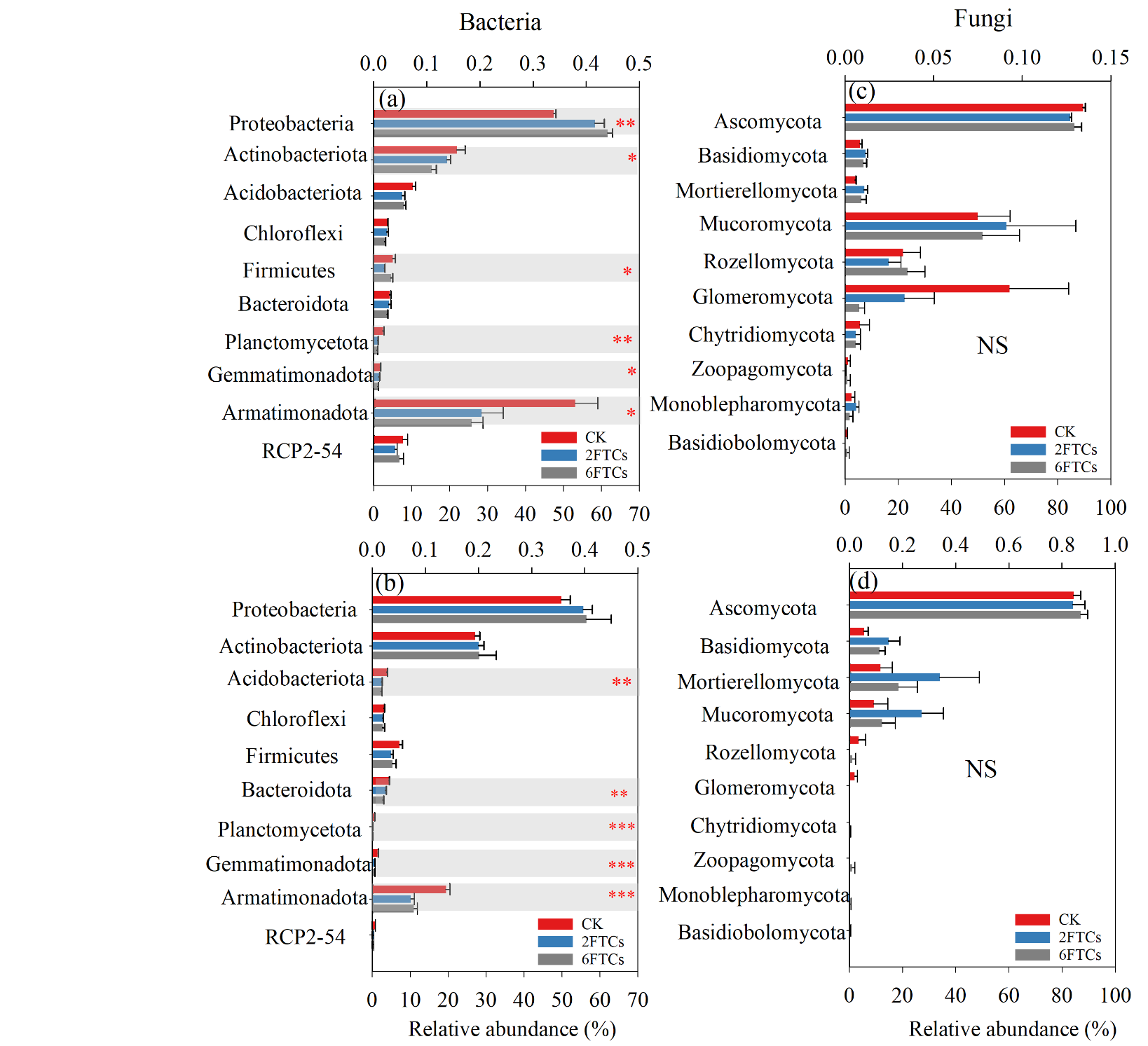
**

**Fig. S4**


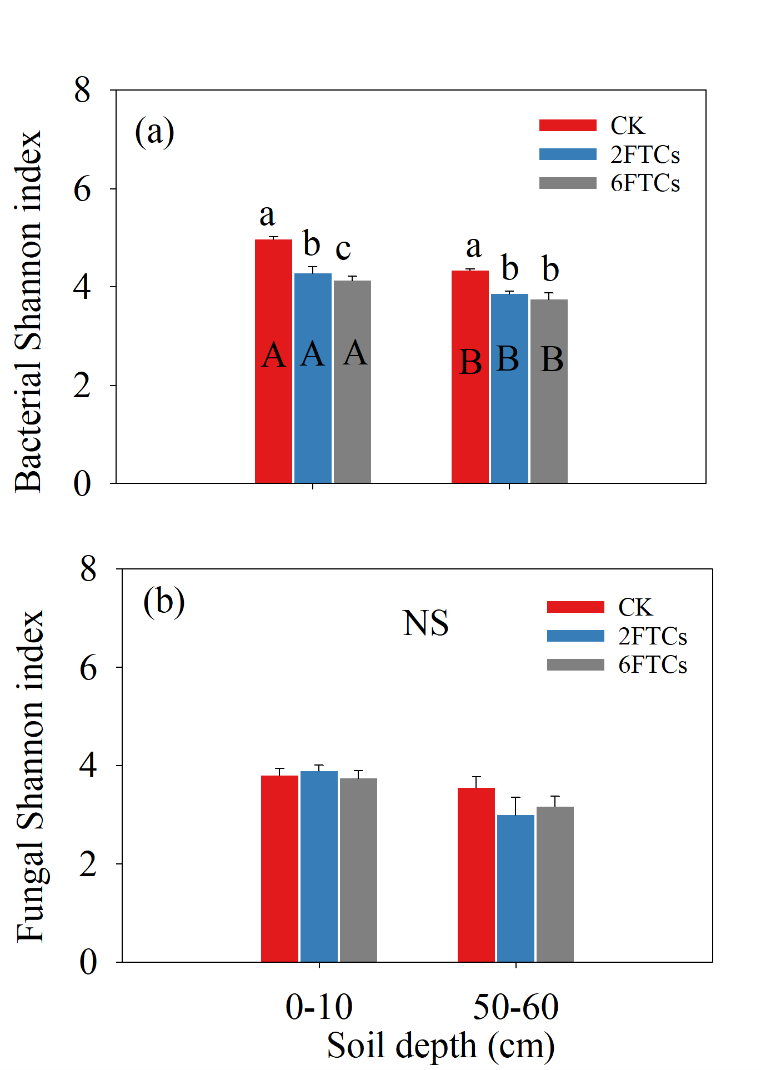

Supplement: Supplementary file 1 [file Data_Sheet_1.docx]
